# Commentary: Preliminary comparison of efficacy and safety between direct bypass surgery and endovascular recanalization therapy in adult ischemic moyamoya disease

**DOI:** 10.3389/fneur.2026.1833721

**Published:** 2026-05-07

**Authors:** Hao Chen, Yichun Zou

**Affiliations:** Department of Neurosurgery, Zhongnan Hospital of Wuhan University, Wuhan, Hubei, China

**Keywords:** bypass, endovascular recanalization, moyamoya disease, restenosis, selection bias

We read with great interest the recent article comparing direct bypass surgery with endovascular recanalization therapy in adult ischemic moyamoya disease (1). The authors should be commended for addressing an important clinical question and providing valuable real-world data in a rare disease setting.

However, several issues warrant careful consideration when interpreting the findings. First, the comparison between treatment groups is likely affected by substantial selection bias. According to the authors treatment algorithm, endovascular recanalization was mainly performed in patients with short-segment occlusion and favorable distal landing zones, whereas direct bypass was more commonly used for patients with more diffuse or complex arterial disease. Because vascular anatomy and disease extent strongly influence both procedural success and clinical outcome in moyamoya disease, such non-random allocation may confound the observed similarity in short-term outcomes. Future studies may benefit from approaches such as propensity score matching or stratified analyses to better address baseline imbalances.

Second, the follow-up duration appears insufficient to evaluate the durability of endovascular recanalization. Moyamoya disease is characterized by progressive intimal hyperplasia and ongoing vascular remodeling (2), processes associated with a high risk of restenosis after angioplasty or stenting. Recent evidence further supports the limited long-term durability of endovascular therapy, with relatively high rates of restenosis and recurrent ischemic events (3, 4). A follow-up of at least 12 months, as commonly adopted in surgical revascularization studies, would be more appropriate to assess long-term vessel patency and clinical outcomes (5, 6).

Third, the pathophysiological rationale for mechanical recanalization in moyamoya disease remains uncertain. Unlike intracranial atherosclerotic stenosis, moyamoya disease represents a progressive occlusive arteriopathy driven by abnormal intimal proliferation. As a result, mechanical dilation or stent placement may have limited ability to address the underlying disease process, although a potential role in highly selected cases, such as short-segment or unilateral lesions, cannot be excluded (7).

Taken together, although the study suggests that endovascular recanalization may be technically feasible in selected patients, the potential for selection bias, limited follow-up duration, and underlying pathophysiological considerations make it difficult to conclude that this strategy provides outcomes comparable to direct bypass surgery. Further prospective studies with longer follow-up will be necessary to clarify the role of endovascular approaches in adult moyamoya disease.
